# JNS Review Recognition 2019

**DOI:** 10.1017/jns.2019.35

**Published:** 2019-11-14

The Editorial Board of the *Journal of Nutritional Science* would like to thank the following for their contribution as peer reviewers in 2019:

Folasade Adebayo
Benjamin Allès
Naser Alsharairi
Rona Antoni
Bernardo Baldisserotto
Marc Bomhof
Andrew Bottley
Maria Bouga
Roger Bouillon
Kathryn Bradbury
Alex Brito
Danyel Bueno-Dalto
Daniela Canella
Monica Carlsen
Lucy Cheke
Raphaël Chouinard-Watkins
Miriam Clegg
Adam Collins
Karen Corbin
Diana Coulon
Marta Crous Bou
Willem De Keyzer
Tim De Meyer
Saskia de Pee
Paraskeui Detopoulou
Georgina Dodd
Lauren Duckworth
Alastair Duncan
Peter Eckl
Samer El-Kadi
Aya Fujiwara
Melawhy Garcia
Yong Meng Goh
Horst Göring
Sueppong Gowachirapant
Peter Grabowski
Neil Harris
Anne Hatloy
Stephen Hennigar
Adela Hruby
Hiroshi Ichikawa
Roberta Imperatore
Arora Ingadottir
Suvi Itkonen
Sandra Iuliano
Hélène Jacques
Xia Jiang
Cyril Kendall
Svyatoslav Komarnytsky
Meron Lewis
Dongmin Liu
Trias Mahmudiono
Emma McMahon
Anine Medin
Jelena Meinila
J. Bernadette Moore
Alyssa Moran
Charlotte Mortensen
Laurent Mosoni
Peter Mount
Anarina Murillo
David Neiman
Tzortzis Nomikos
Signe Okkels
Chad Paton
Jaqueline Pereira
Julia Peterson
Krista Power
Damar Pramusinto
Anabelle Retondario
Caroline Richard
Christian Ritz
Asman Sadino
Rodrigo San-Cristobal
Jonathan Scott
Cecilia Severi
Harbindar Singh
Kylie Smith
Daniel So
Michelle Swainson
Carmen Tekwe
Adriana van Ballegooijen
Corné van Dooren
María Victoria Velarde
Joseph Wakshlag
Linlin Wang
Lynn Weber
Jean Welsh
Emma Wightman
Hiroshi Yokomichi
Satoko Yoneyama

